# The impact of the Khartoum war on dental education

**DOI:** 10.11604/pamj.2024.48.119.44219

**Published:** 2024-07-19

**Authors:** Nada Tawfig Hashim, Vivek Padmanabhan, Mariam Elhadi Elsheikh, Bakri Gobara Gismalla, Mohammed Mustahsen Rehman

**Affiliations:** 1 Department of Periodontics, RAK College of Dental Sciences, RAK Medical and Health Sciences University, Ras Al-Khaimah, United Arab Emirates,; 2 Department of Pediatric and Preventive Dentistry, RAK College of Dental Sciences, RAK Medical and Health Sciences University, Ras Al-Khaimah, United Arab Emirates,; 3 Department of Oral and Maxillofacial, Faculty of Dentistry, University of Khartoum, Khartoum, Sudan,; 4 Department of Oral Rehabilitation, Faculty of Dentistry, University of Khartoum, Khartoum, Sudan

**Keywords:** Khartoum war, conflict, dental education, students

## Abstract

The relentless conflict in Khartoum has severely crippled higher education, especially in specialized fields like dental education. The war has wreaked havoc on academic schedules, severely damaging infrastructure, and creating an atmosphere of pervasive uncertainty and stress for students and faculty alike. Academic continuity has been shattered, with courses and training programs facing frequent disruptions, leaving students struggling to complete their studies and gain essential practical experience. Faculty members, too, are under immense pressure, dealing with the dual burdens of maintaining educational standards and ensuring personal safety. This commentary delves into the devastating and far-reaching consequences of the ongoing conflict on dental education in Khartoum, highlighting the urgent need for comprehensive recovery and support strategies to restore this critical field.

## Essay

Sudan is currently engulfed in a grave humanitarian crisis triggered by intense armed conflicts that erupted on April 15^th^ between the Sudanese Armed Forces (SAF) and the Rapid Support Forces (RSF). The conflicts span various locations, including the capital city of Khartoum, North Kordofan, Darfur, and River Nile provinces [1]. The Ministry of Health's report dated May 9^th^, 2023 highlighted the severe toll on civilians, with 550 fatalities, 4926 injuries, and over one million people displaced from their homes. While the direct human cost is apparent, it is crucial to explore the less visible but significant impacts on the dental education system, both in the immediate and long-term contexts [2].

The Khartoum War, part of a broader conflict scenario in Sudan, has posed immense challenges to several societal sectors, including education [3]. Dental education, in particular, faces heightened vulnerability due to its reliance on specialized infrastructure, equipment, and hands-on clinical training [4]. This article aims to elucidate the multifaceted impacts of the Khartoum war on dental education, encompassing the destruction of material assets and the intangible effects on academic progress and community confidence. By investigating these factors, we seek to understand the extent of the harm caused by the war and the resilience and ingenuity demonstrated by the academic and healthcare communities in Khartoum and beyond.

### Background of dental education in Sudan

Before establishing the first dental school, Sudan's oral health services were rudimentary and disorganized. The necessity for a well-established institution to produce dentists with specialized education became evident to meet the growing healthcare needs of the population. A pivotal moment in Sudanese dental education occurred when the first dental school was established [5]. Dental education in Sudan commenced in the mid-20^th^ century when the government recognized the need to create national programs for educating and training dental professionals. By the late 1970s, Sudan had established its first dental school, aiming to cultivate a self-reliant workforce of domestically educated professionals capable of efficiently addressing the nation's oral health needs. Before this, Sudan had relied on dentists trained abroad [5].

The Faculty of Dentistry at the University of Khartoum was the first institution in Sudan to provide dental education, founded to meet the pressing needs of the nation's people through advancing dental education and oral healthcare. This institution was established in response to the increasing need for structured dental education in Sudan [6]. The University of Khartoum made significant progress by creating this faculty, acknowledging the crucial role of oral health in the broader healthcare system. The initial challenges included a scarcity of skilled instructors, the need for a comprehensive curriculum adhering to international standards, and the creation of well-equipped facilities for hands-on instruction. The establishment of the Faculty of Dentistry in 1970 marked a major advancement in Sudan's dental education [7]. This and subsequent institutions were equipped with essential academic and clinical resources to provide comprehensive dental education, including fundamental scientific coursework and advanced clinical training. The rise of dental schools in Sudan reflects the government's resolve to enhance oral health and education standards. Multiple factors essential for understanding the growth of dental healthcare education in the area influenced the subsequent expansion after the first institution was established [8]. Healthcare education in Sudan faces unique challenges and opportunities due to the country's extensive geographical area and diverse population distribution. Effectively catering to different populations necessitates considering geographical obstacles and demographic discrepancies, which have driven the establishment of institutions in various regions [9]. The decentralized dental education approach involves setting up schools in both urban and rural areas, aiming to tackle disparities in regional dental education and care access. However, decentralization poses challenges such as maintaining uniform educational standards, providing quality assurance, and efficiently managing resources [10].

### Pre-conflict situation in dental education

Before the conflict, Sudan had developed educational institutions providing dental education, with the University of Khartoum playing a significant role through its Faculty of Dentistry. This institution was renowned for its extensive educational offerings, particularly in the medical domain. Research conducted in Khartoum revealed numerous dental colleges in the capital city, indicating a network of educational possibilities in this field [11].

Political and societal instability, such as wars, can significantly impact educational institutions, disrupting academic calendars, damaging buildings, and displacing students and staff [12]. Dental education in war zones often encounters substantial difficulties during and after instability, similar to other educational sectors. Before the Khartoum War, Sudan had achieved notable advancements in expanding its dental education sector. Many colleges in Khartoum and other cities provided dental programs, leading to a progressive rise in locally educated dental professionals. These programs were distinguished by a curriculum combining academic knowledge with practical clinical skills, preparing students to address the varied oral health requirements of the Sudanese population [13].

Partnerships with overseas dental schools and organizations enhanced Sudanese dental education, offering professors and students valuable opportunities for exchange and ongoing education. Despite budget constraints, research played a crucial role in Sudan's dental education, focusing on topics directly related to the country's health challenges.

### Immediate effects of the Khartoum war on dental education

**Destruction of infrastructure and loss of educational materials:** the battle in Khartoum caused substantial damage to educational facilities, including dental schools and clinics. Structures were demolished or extensively damaged, and essential educational resources, such as textbooks, laboratory equipment, and dental tools, were lost. This physical devastation had an immediate and significant impact on the institutions' capacity to provide education and training [14,15].

**Displacement of faculty and students:** the conflict resulted in the forced relocation of many individuals, including students and staff members of dental schools. This displacement disrupted the continuity of education for numerous students and resulted in a scarcity of teaching personnel, further straining the ability of dental schools to function efficiently [1].

**Interruption of academic schedules and clinical training:** the conflict necessitated the cancellation of academic schedules, including lectures, examinations, and crucially, clinical training sessions. Clinical training, a fundamental aspect of dental education, was significantly impacted due to the destruction of clinical facilities and the hazardous conditions in conflict zones. The suspension of clinical training not only delayed students' education but also undermined the competence of future dental practitioners [16].

### Effect of conflict on the mental health of dental students

The Khartoum conflict has had a complex impact on the mental well-being of dental students, as wars are known to exert significant psychological burdens on students. Conflict situations often exacerbate mental health problems, including stress, anxiety, and depression, which are already prevalent among dental students due to the rigorous nature of their education [17].

Students living in conflict-affected regions may encounter various mental health issues. Individuals exposed to conflict may be more vulnerable to post-traumatic stress disorder, depression, and anxiety. Additionally, their overall quality of life may be diminished, even after the conflict has ended. The pressure of adapting to changes in their educational environment, concerns for personal safety, and worries about the future can contribute to their psychological distress [18,19].

Research on dental students' well-being has shown that even without additional stressors like conflicts, dental students often experience significant psychological stress. Factors contributing to this stress include the challenging curriculum, the need for precision in clinical practice, concerns about patient care, exams, and future job prospects [20-22].

Furthermore, the use of distance learning, initially introduced as a response to the COVID-19 pandemic and potentially applicable in conflict scenarios, can impact dental students' mental well-being. The lack of in-person interactions, the difficulty in acquiring practical therapeutic skills, and the overall disruption to traditional educational methods can lead to feelings of isolation and increased stress levels [23,24].

Acknowledging that these insights are broad and derived from existing research is essential. The overall impact of the Khartoum war on dental students would likely align with these patterns, but there may also be unique elements influenced by the specific conditions of the conflict. Targeted mental health interventions for students affected by violence in resource-constrained environments are crucial but often inadequate, highlighting the need for increased emphasis on the mental well-being of these students. To provide comprehensive support, educational institutions, health services, and the community must collaborate to offer a range of resources, counseling, and other forms of psychological support to students affected by conflict [25].

### Adaptive measures and innovations in dental education

During the early stages of the April 15 violence in Sudan, a significant number of medical facilities in conflict zones were targeted and attacked. Administrative facilities were affected in 41.2% of cases, endangering crucial documentation systems, student data, and college servers. University data were particularly vulnerable if not stored on an internet-based storage system or cloud. Despite facing challenges, about 60.3% of the schools managed to resume some instructional activities by implementing measures such as online education and establishing collaborations with both local and international universities. Notwithstanding these difficulties, the dental education community in Khartoum and Sudan as a whole has demonstrated resilience and flexibility [11].

**Shifting to online platforms and modular courses *:*** in response to the necessity of continuing education amid the physical devastation of facilities, certain institutions adopted modular courses and online learning platforms. This transition enabled students to advance in their theoretical studies despite being displaced or residing in conflict-affected regions [11].

**International aid and partnerships:** the global dental education community contributed by donating materials, funding, and online learning resources. International partnerships facilitated virtual clinical training sessions and webinars, filling the gap left by the disruption of in-person training [26].

**Case studies of resilience and adaptation *:*** specific examples of adaptation include a dental school that transitioned to fully online lectures within weeks of the conflict's escalation and another that established a network of volunteer alumni to offer remote mentorship and clinical guidance to students. Although these adaptive measures have not been without their challenges, they have ensured the continuation of dental education under exceptionally difficult circumstances. Additionally, they provide valuable insights into the importance of community, resilience, and flexibility in the face of adversity [11].

### Long-term consequences for dental education

**Brain drain:** the crisis in Khartoum has accelerated the phenomenon of brain drain, as highly qualified academics and potential graduates seek safety and security in other countries. The departure of skilled individuals exacerbates the existing challenges in dental education, lowering the standard of instruction and limiting the potential for research and innovation. The loss of these individuals reduces the current number of dental professionals and impacts the next generation of educators and practitioners in Sudan [27].

**Economic impact:** the economic strain on dental education is profound. The costs associated with rebuilding damaged infrastructure, replacing lost educational materials, and adapting to new delivery methods are substantial. Additionally, the overall economic downturn caused by the conflict reduces the availability of funding for education, leading to budget cuts and further compromising the quality of dental education [28].

**Changes in curriculum:** the conflict has necessitated a reassessment of the dental curriculum to align more effectively with the altered realities. There is a potential trend towards prioritizing emergency and conflict-related healthcare, explicitly addressing urgent oral health issues that arise during conflicts. While this adaptability is crucial, it may lead to the neglect of broader educational objectives, impacting the comprehensive preparation of future dental practitioners.

### The role of dental professionals in post-conflict recovery

Dental professionals play a crucial role in the recovery and reconstruction of areas affected by conflict. In addition to providing essential dental care, they can contribute significantly to public health efforts, such as disease prevention programs and community health education. Their involvement in humanitarian efforts addresses immediate health needs and helps restore community trust, laying the foundation for long-term recovery and stability [29] Table 1.

**Table 1:** summarizes the various roles that dental professionals can play in the post-conflict recovery process in Sudan, highlighting the potential impacts of their involvement in different areas

Role	Description	Impact
Providing Essential Dental Care	Dental professionals offer necessary dental services to communities.	Immediate relief for individuals with dental issues.
		Helps prevent oral health problems from worsening, thereby improving overall health and quality of life.
Public Health Efforts	Participation in public health initiatives, including disease prevention programs.	Contributes to broader public health goals.
		Promotes awareness and prevention of common oral health issues.
		Enhances community health through education and preventive care.
Community Health Education	Educating the community about oral health and hygiene practices.	Empower communities with knowledge to maintain good oral health.
		Reduces the prevalence of preventable oral health conditions.
		Strengthens community resilience and self-reliance in health matters.
Humanitarian Efforts	Involvement in humanitarian initiatives to address immediate health needs.	Provides critical health support in post-conflict settings.
		Restores trust in healthcare systems.
		Demonstrates the commitment of dental professionals to community well-being.
Restoring Community Trust	Building trust in the healthcare system through consistent and compassionate care.	Encourages community engagement with health services.
		Rebuilds the reputation of healthcare institutions.
		Supports the healing and recovery process by showing care and reliability.
Participating in Recovery Programs	Engagement in programs aimed at rebuilding healthcare infrastructure and services.	Helps restore damaged dental facilities.
		Contributes to the overall recovery and strengthening of the healthcare system.
		Ensures sustainable improvements in health service delivery post-conflict.
Supporting Mental Health	Addressing the psychological impacts of conflict through support and care.	Provides holistic care that includes mental well-being.
		Helps mitigate the psychological distress caused by conflict.
		Encourages a more comprehensive approach to health recovery.
Training and Education	Training new dental professionals and providing ongoing education to existing practitioners.	Ensures a continuous supply of skilled dental professionals.
		Keeps practitioners updated with the latest knowledge and techniques.
		Enhances the quality and effectiveness of dental care services.
Research and Development	Researching to understand and address the unique health challenges in post-conflict settings.	Generates evidence-based practices tailored to post-conflict recovery.
		Contributes to the global knowledge base on health recovery in conflict-affected areas.
		Drives innovation and improvements in dental care.
Collaboration with International Partners	Working with global institutions and organizations to support recovery efforts.	Leverages international resources and expertise.
		Facilitates the exchange of knowledge and best practices.
		Strengthens local capacity through partnerships and collaborations.
Policy Advocacy	Advocating for policies that support health recovery and resilience.	Influences health policy to prioritize recovery efforts.
		Secures funding and resources for rebuilding healthcare infrastructure.
		Ensures that dental health is included in broader health recovery and development plans.

### Future prospects for dental education in Khartoum

Despite the challenges, there are opportunities for growth and advancement in dental education in Khartoum. The experiences of adapting to conflict situations have underscored the importance of resilience and flexibility in education. To improve the quality of dental education, it is crucial to make strategic investments in infrastructure, technology, and curriculum development. This can be achieved by examining how dental curriculums can adapt to address the conflict. Specifically, it is essential to include training on emergency and disaster response, consider mental health in dental care, and adjust educational goals to align with the evolving health situation in Sudan [1].

Partnerships with international institutions and organizations can provide support and resources, facilitating recovery and progress. Case studies can showcase examples of effective collaboration, highlighting the types of assistance provided, such as faculty exchange programs, donations of materials, and funding for infrastructure rebuilding. These collaborations have had a significant impact on the continuation of dental education [30,31].

Moreover, developing strategies to retain skilled professionals within the country and attract experts from the diaspora can help mitigate the negative effects of brain drain. Ensuring the stability and safety of educational environments and offering incentives and opportunities for professional growth are essential. This may involve considering potential governmental and institutional measures, such as increased allocation of resources to education, incentives for professionals to stay or return, and establishing research and development opportunities within the country [29].

Implementing support systems and mental health services in dental schools is crucial to promoting the well-being of all members of the academic community, especially given the profound psychological implications of the conflict for dental students and faculty, including stress, anxiety, and challenges in maintaining educational motivation [32] Table 2.

**Table 2:** provides a structured overview of the psychological implications of the conflict on dental students and faculty, along with suggested measures to support their mental well-being

Aspect	Description	Impact	Suggested Measures
**Psychological Implications**	The conflict has caused significant stress, anxiety, and other mental health issues among dental students and faculty.	Increased levels of psychological distress, including stress and anxiety.	
		Challenges in maintaining educational motivation and focus.	
		Potential long-term effects on mental well-being and academic performance.	
**Stress**	Dental students and faculty experience heightened stress due to the instability and uncertainty caused by the conflict.	Decreased ability to concentrate on studies and teaching.	
		Heightened emotional and physical strain.	
**Anxiety**	Anxiety levels have increased among students and faculty due to safety concerns, displacement, and disruption of normal routines.	Negative impact on mental health and overall well-being.	
		Difficulty in coping with academic and personal challenges.	
**Educational Motivation**	Maintaining motivation for educational activities is challenging in a conflict-affected environment.	Lower academic performance.	
		Increased dropout rates and reduced participation in academic activities.	
**Support Systems and Services**	Implementing mental health support systems and services in dental schools is crucial to address the psychological impact of the conflict.	- Improved mental well-being and resilience among students and faculty.	
		Better coping mechanisms for dealing with stress and anxiety.	
		Enhanced academic performance and motivation.	
**Counseling Services**	Providing access to professional counseling services to help students and faculty cope with psychological distress.	Reduced levels of stress and anxiety.	Establish dedicated counseling centers.
		Better mental health support for those affected by the conflict.	Train counselors to address conflict-related mental health issues.
		Creation of a supportive environment within the academic community.	
**Peer Support Programs**	Establishing peer support programs where students and faculty can support each other through shared experiences and mutual understanding.	Increased sense of community and support.	Create peer support groups and networks.
		Improved coping strategies through peer interactions.	Encourage open communication and sharing of experiences.
		Reduction in feelings of isolation.	
**Stress Management Workshops**	Conducting workshops and seminars on stress management techniques and coping strategies.	Enhanced ability to manage stress and anxiety.	Organize regular workshops on stress management.
		Improved mental resilience and well-being.	Provide resources and materials on coping strategies.
		Practical tools and techniques for coping with academic and personal stressors.	
**Mental Health Awareness Campaigns**	Raising awareness about mental health issues and the importance of seeking help through campaigns and educational programs.	Increased awareness and understanding of mental health issues.	Launch mental health awareness campaigns.
		Encouragement to seek help and support.	Integrate mental health education into the curriculum.
		Reduction in stigma associated with mental health problems.	
**Faculty Training Programs**	Training faculty members to recognize and address mental health issues among students and provide appropriate support and referrals.	Enhanced ability of faculty to support students.	Develop faculty training modules on mental health.
		Early identification and intervention for students facing mental health challenges.	Conduct regular training sessions for faculty members.
		Creation of a supportive academic environment.	
**Flexible Academic Policies**	Adopting flexible academic policies to accommodate the needs of students affected by the conflict, such as extended deadlines, alternative assessment methods, and leaves of absence.	Reduced academic pressure on students.	Implement flexible policies for deadlines and assessments.
		Increased ability to balance academic and personal challenges.	Provide options for leaves of absence.
		Improved retention and success rates for students affected by the conflict.	

## Conclusion

In conclusion, the Khartoum War has profoundly disrupted dental education, but the remarkable resilience and adaptability of the academic community offer a beacon of hope. By learning from these experiences and prioritizing recovery and development, the future of dental education in Khartoum can shine brightly, playing a crucial role in enhancing the overall health and well-being of the Sudanese population. It is imperative that peacebuilding efforts urgently focus on restoring educational services and providing robust support mechanisms for mental health to mitigate the long-term repercussions of the conflict on Khartoum's dental profession.
